# High pH Alleviated Sweet Orange (*Citrus sinensis*) Copper Toxicity by Enhancing the Capacity to Maintain a Balance between Formation and Removal of Reactive Oxygen Species and Methylglyoxal in Leaves and Roots

**DOI:** 10.3390/ijms232213896

**Published:** 2022-11-11

**Authors:** Jiang Zhang, Xu-Feng Chen, Wei-Lin Huang, Huan-Huan Chen, Zeng-Rong Huang, Xin Ye, Li-Song Chen

**Affiliations:** College of Resources and Environment, Fujian Agriculture and Forestry University, Fuzhou 350002, China

**Keywords:** antioxidant enzyme, Cu, Cu–pH interactions, glyoxalase, metallothioneins, MG and ROS detoxification, phytochelatins, sulfur-metabolism-related enzyme

## Abstract

The contribution of reactive oxygen species (ROS) and methylglyoxal (MG) formation and removal in high-pH-mediated alleviation of plant copper (Cu)-toxicity remains to be elucidated. Seedlings of sweet orange (*Citrus sinensis*) were treated with 0.5 (non-Cu-toxicity) or 300 (Cu-toxicity) μM CuCl_2_ × pH 4.8, 4.0, or 3.0 for 17 weeks. Thereafter, superoxide anion production rate; H_2_O_2_ production rate; the concentrations of MG, malondialdehyde (MDA), and antioxidant metabolites (reduced glutathione, ascorbate, phytochelatins, metallothioneins, total non-protein thiols); and the activities of enzymes (antioxidant enzymes, glyoxalases, and sulfur metabolism-related enzymes) in leaves and roots were determined. High pH mitigated oxidative damage in Cu-toxic leaves and roots, thereby conferring sweet orange Cu tolerance. The alleviation of oxidative damage involved enhanced ability to maintain the balance between ROS and MG formation and removal through the downregulation of ROS and MG formation and the coordinated actions of ROS and MG detoxification systems. Low pH (pH 3.0) impaired the balance between ROS and MG formation and removal, thereby causing oxidative damage in Cu-toxic leaves and roots but not in non-Cu-toxic ones. Cu toxicity and low pH had obvious synergistic impacts on ROS and MG generation and removal in leaves and roots. Additionally, 21 (4) parameters in leaves were positively (negatively) related to the corresponding root parameters, implying that there were some similarities and differences in the responses of ROS and MG metabolisms to Cu–pH interactions between leaves and roots.

## 1. Introduction

As a cofactor for many enzymes, copper (Cu) participates in various physiological processes of plants, including respiration, photosynthesis, protection against oxidative damage, and carbohydrate and nitrogen metabolisms [1,2,3,4,5,6,7]. Cu is also a naturally occurring heavy metal in soil and is highly toxic to crops when excessive concentration is applied. Cu excess will affect soil fertility, inhibit crop growth, reduce crop yield and quality, and even threaten human health [8,9,10,11]. In some old *Citrus* orchards, Cu excess in soil is a major factor that reduces productivity and quality of *Citrus* due to human activities, such as heavy and long-term application of Bordeaux mixture, wastewater irrigation, urban compost, mining, and industrialization [11,12,13,14]. 

The phytotoxicity of Cu depends greatly on soil pH [15]. Previous reports indicated that high pH had antagonistic actions against the harmful effects of Cu toxicity on plants [2,3,16,17]. However, most researchers have focused on examining interactive effects of Cu–pH on plant biomass, root morphology, root exudates, uptake of nutrient and water, and leaf pigments, photosynthesis, non-structural carbohydrates, gene expression, metabolite abundances, and cell wall components [2,3,16].

In plants, excessive Cu can increase reactive oxygen species (ROS) and methylglyoxal (MG) formation and overaccumulation via Haber–Weiss reaction and/or Fenton reaction, resulting in oxidative damage and even cell death [18,19,20,21,22]. ROS can be scavenged by non-enzymatic (ascorbate (ASC), metallothioneins (MTs), phytochelatins (PCs) and reduced glutathione (GSH)) and enzymatic (sulfur-metabolism-related enzymes and antioxidant enzymes) systems [23]. Glyoxalase (Gly) I and Gly II are the main actors in MG removal. Coordinated actions of ROS and MG detoxification systems are very crucial to managing the enhanced formation of ROS and MG [24]. Although Cu-toxic impacts on ROS and MG formation and scavenging have been investigated in some detail [20,25], reports on Cu–pH-interaction-induced alterations of ROS and MG metabolisms are rare. Recently, Zhang et al. [2] observed that elevated pH could reduce superoxide anion production rate (SAPR) and malondialdehyde (MDA) concentration in Cu-toxic sweet orange (*Citrus sinensis*) leaves. Unfortunately, this study examined only the two parameters involved in ROS and MG metabolisms. In rice (*Oryza sativa*), GSH- or ASC-mediated mitigation of Cu toxicity involved reduced oxidative damage by lessening ROS levels and increasing antioxidant enzyme activities in leaves [25]. In cucumber (*Cucumis sativus*), melatonin could alleviate Cu toxicity via enhancing Cu sequestration in vacuoles due to increased accumulation of GSH and PCs and preventing oxidative damage due to decreased ROS generation and increased antioxidant systems [26]. Abdulmajeed et al. [21] reported that acetylsalicylic-acid- and NO-mediated mitigation of *Vigna radiata* Cu toxicity involved increased activities of glutathione reductase (GR), catalase (CAT), ASC peroxidase (APX), superoxide dismutase (SOD), Gly I and Gly II, and levels of ASC and GSH and decreased levels of H_2_O_2_, superoxide anion, MG, and MDA in Cu-toxic leaves. Shan et al. [27] found that H_2_S protected wheat (*Triticum aestivum*) seedlings against Cu-toxicity through the reduction in oxidative damage (MDA) due to increased activities of GR, dehydroascorbate (DHA) reductase (DHAR), monodehydroascorbate (MDHA) reductase (MDHAR), APX, and concentrations of GSH and ASC in leaves. Thus, elevated pH might reduce Cu-toxicity-induced oxidative damage through coordinated actions of ROS and MG detoxification systems, thus protecting plants against Cu toxicity. 

*Citrus* are widely planted in acidic soils in southern China with high concentrations and bioavailability of Cu and are vulnerable to Cu toxicity [13,28,29]. In this paper, we systematically examined interactive impacts of Cu–pH on ROS and MG metabolisms in sweet orange leaves and roots in order to test the hypothesis that high pH-mediated mitigation of sweet orange Cu toxicity involved enhanced capacity to maintain a balance between ROS and MG formation and removal in roots and leaves and to understand the different responses of leaf and root ROS and MG metabolisms to Cu–pH interactions.

## 2. Results

### 2.1. Impacts of Cu–pH Interactions on Seedling Growth, SAPR, H_2_O_2_ Production Rate (HPR), MDA, and MG in Leaves and Roots

Increased pH alleviated the inhibitory actions of Cu toxicity on root and shoot growth. Without Cu toxicity, only pH 3.0 (low pH) slightly decreased shoot and root growth. With Cu toxicity, low pH greatly inhibited shoot and root growth (Figure S1).

**Figure 1:**
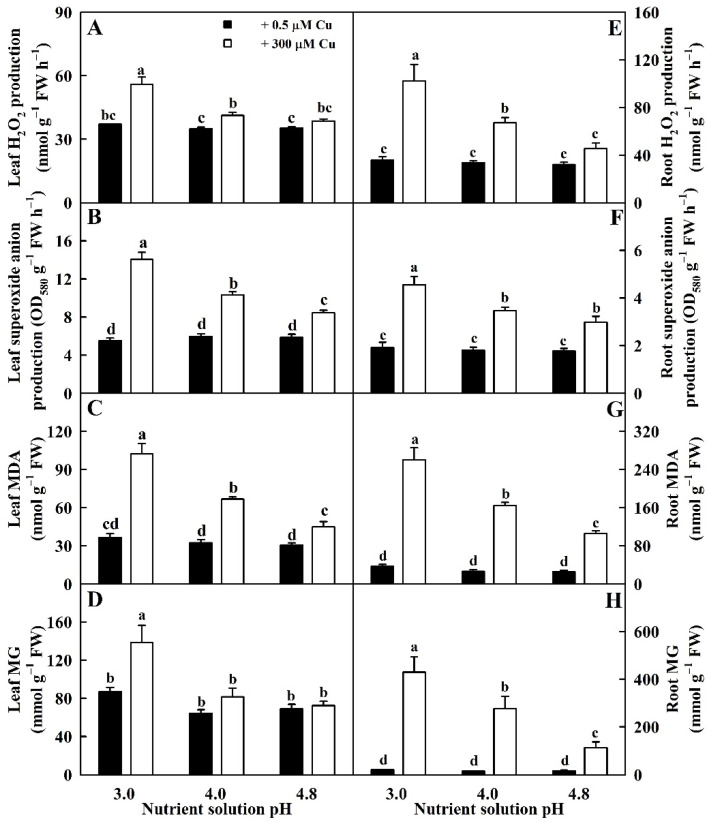
Impacts of Cu–pH interactions on HPR (**A**,**E**), SAPR (**B**,**F**), MDA (**C**,**G**), and MG (**D**,**H**) concentrations in leaves and roots. Bars represent means ± SE (*n* = 4). Different letters above the bars indicate a significant difference at *p* < 0.05.

Cu toxicity elevated HPR, SAPR, MDA, and MG concentrations in leaves and roots, especially in low pH, except for unaltered HPR and MG concentrations in pH-4.8-treated leaves and MG concentration in pH-4.0-treated leaves. HPR, SAPR, MDA, and MG concentrations were elevated by low pH in 300 μM Cu-treated leaves (LCu300) and 300 μM Cu-treated roots (RCu300), but unaltered in 0.5 μM Cu-treated leaves (LCK) and 0.5 μM Cu-treated roots (RCK) (Figure 1). 

### 2.2. Impacts of Cu–pH Interactions on the Activities of Enzymes Involved in ROS and MG Removal in Leaves and Roots

**Figure 2:**
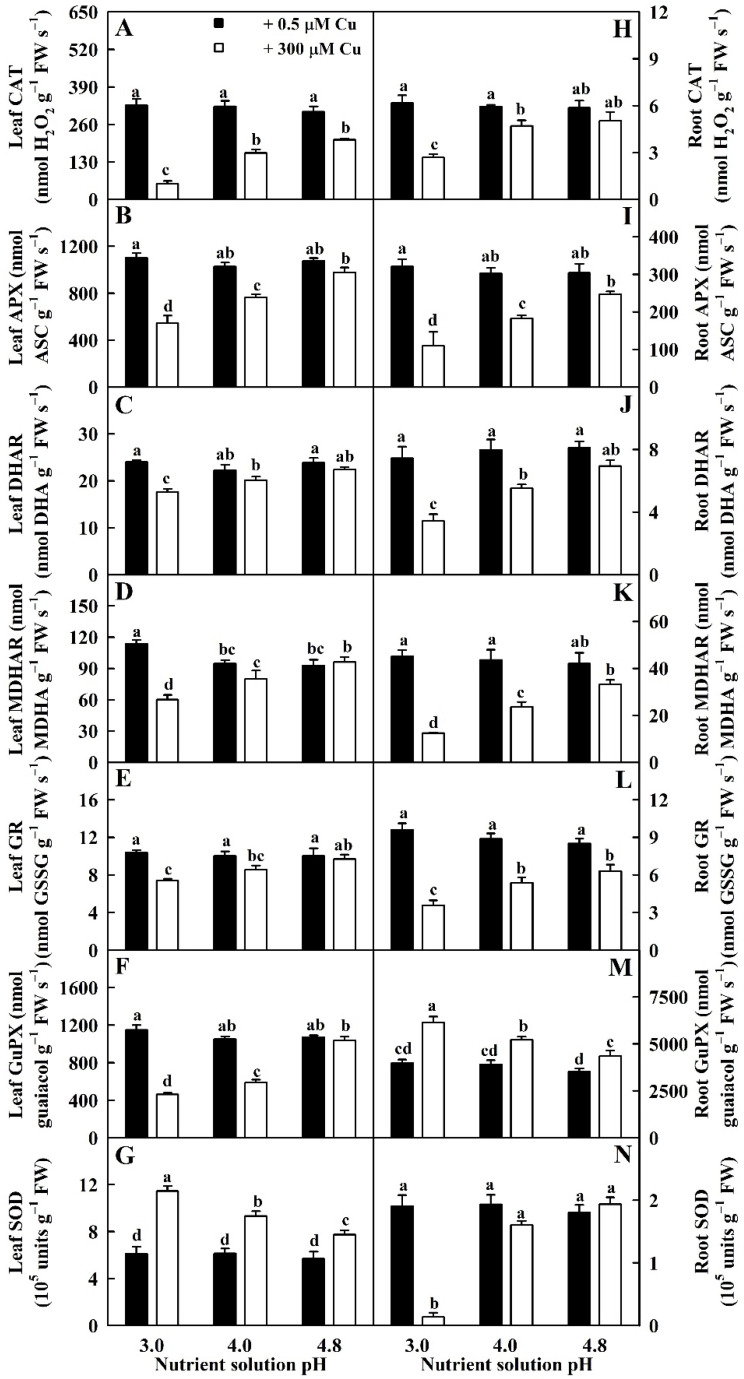
Impacts of Cu–pH interactions on the activities of CAT (**A**,**H**), APX (**B**,**I**), DHAR (**C**,**J**), MDHAR (**D**,**K**), GR (**E**,**L**), GuPX (**F**,**M**), and SOD (**G**,**N**) in leaves and roots. Bars represent means ± SE (*n* = 4). Different letters above the bars indicate a significant difference at *p* < 0.05.

Cu toxicity reduced the activities of DHAR, APX, MDHAR, CAT, and GR in leaves and roots and guaiacol peroxidase (GuPX) in leaves and SOD in roots at pH 3.0 and pH 4.0 except for unaltered activities of DHAR and MDHAR in pH-4.0-treated leaves and SOD in pH 4.0-treated roots. However, it had no impacts on their activities in pH-4.8-treated leaves and roots with the exceptions that Cu-toxicity slightly reduced the activities of GR in roots and CAT in leaves. However, low pH enhanced the activities of SOD in LCu300 and GuPX in RCu300 (Figure 2).

The activities of DHAR, APX, MDHAR, CAT and GR in LCu300 and RCu300, GuPX in LCu300 and SOD in RCu300 increased with the increase in pH. The activities of the seven antioxidant enzymes in LCK and RCK were not significantly altered by the change in pH except for a slight increase in MDHAR activity in LCK at pH 3.0 (Figure 2).

**Figure 3:**
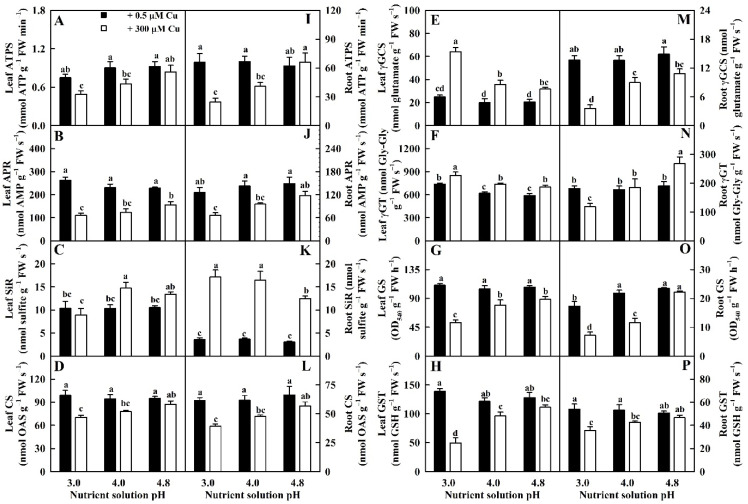
Impacts of Cu–pH interactions on the activities of ATPS (**A**,**I**), APR (**B**,**J**), SiR (**C**,**K**), CS (**D**,**L**), γGCS (**E**,**M**), γGT (**F**,**N**), GS (**G**,**O**), and GST (**H**,**P**) in leaves and roots. Bars represent means ± SE (*n* = 4). Different letters above the bars indicate a significant difference at *p* < 0.05. Gly-Gly, glycylglycine; OAS, O-acetylserine.

We found that Cu toxicity reduced the activities of ATP sulfurylase (ATPS), adenosine 5′-phosphosulphate reductase (APR), cysteine synthase (CS), glutamine synthetase (GS), and glutathione S-transferase (GST) in leaves and roots and γ-glutamylcysteine synthetase (γGCS) in roots more at pH 3.0 than at pH 4.8. We also found that Cu toxicity did not alter the activities of ATPS, CS, and GST in leaves and roots or APR and GS in roots at pH 4.8. However, Cu toxicity increased or did not significantly influence the activities of sulfite reductase (SiR) and γ-glutamyltransferase (γGT) in leaves and roots and γGCS in roots except for reduced γGT activity in RCu300 at pH 3.0 (Figure 3).

Low pH reduced the activities of ATPS, APR, CS, GS, and GST in LCu300 and RCu300; SiR in LCu300; and γGCS and γGT in RCu300; but it increased the activities of SiR, γGCS, and γGT in LCu300 relative to pH 4.8. The activities of the eight sulfur metabolism-related enzymes in LCK and RCK were not significantly altered by the alteration of pH except for elevated γGT activity in LCK at pH 3.0 and reduced GS activity in RCK at pH 3.0 (Figure 3).

**Figure 4:**
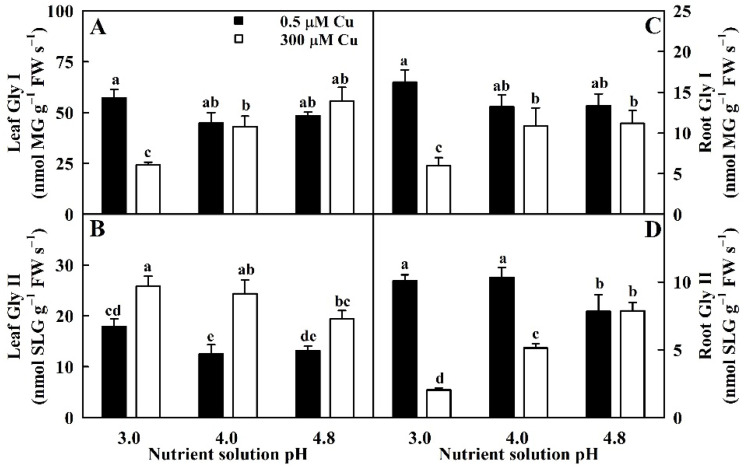
Impacts of Cu–pH interactions on the activities of Gly I (**A**,**C**) and Gly II (**B**,**D**) in leaves and roots. Bars represent means ± SE (*n* = 4). Different letters above the bars indicate a significant difference at *p* < 0.05. SLG, S-D-lactoylglutathione.

Cu toxicity increased Gly II activity in leaves but inhibited or did not affect the activities of Gly I in leaves and roots and Gly II in roots (Figure 4).

Low pH enhanced Gly II activity in LCu300 but inhibited the activities of Gly I in LCu300 and RCu300 and Gly II in RCu300 relative to pH 4.8. The activities of Gly I and Gly II in LCK and RCK remained stable with the alteration of pH except for slightly increased Gly II activity in pH-3.0-treated leaves and slightly decreased Gly II activity in pH-4.8-treated roots (Figure 4). 

### 2.3. Impacts of Cu–pH Interactions on the Concentrations and Ratios of Antioxidant Metabolites Involved in ROS and MG Scavenging in Leaves and Roots

**Figure 5:**
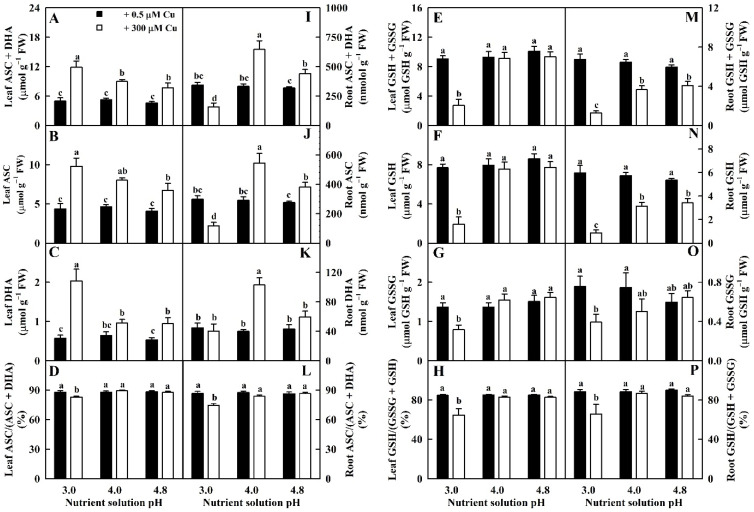
Impacts of Cu–pH interactions on TA (sum of ASC and DHA; (**A**,**I**)), ASC (**B**,**J**) and DHA (**C**,**K**) concentrations; ASC redox state (ASC/TA ratio; (**D**,**L**)); TG (sum of GSH and GSSG; (**E**,**M**)), GSH (**F**,**N**) and GSSG (**G**,**O**) concentrations; and GSH redox state (GSH/TG ratio; (**H**,**P**)) in leaves and roots. Bars represent means ± SE (*n* = 4). Different letters above the bars indicate a significant difference at *p* < 0.05.

Cu toxicity elevated total ascorbate (TA, sum of ASC and DHA), ASC, and DHA concentrations in leaves more at pH 3.0 than at pH 4.0–4.8. Cu toxicity enhanced TA, ASC, and DHA levels in roots more at pH 4.0 than at pH 4.8, but it reduced TA and ASC levels and did not affect DHA levels in pH-3.0-treated roots. Compared to pH 4.8, low pH increased TA, ASC, and DHA concentrations in LCu300 relative to pH 4.8 while repressing their concentrations in RCu300 except for a similar DHA concentration. TA, ASC, and DHA concentrations in LCK and RCK remained unchanged at pH 3.0–4.8. ASC redox state (ASC/TA ratio) in leaves and roots did not differ between Cu–pH combinations except for a drop in pH 3.0 + 300 μM Cu (Figure 5).

Total glutathione (TG, sum of GSH and oxidized glutathione (GSSG)) and GSH levels in leaves and GSSG levels and GSH redox state (GSH/TG ratio) in leaves and roots were similar between Cu–pH combinations except for a drop under pH 3.0 + 300 μM Cu. Cu toxicity reduced TG and GSSG levels in roots. Low pH lowered TG and GSSG levels in RCu300 relative to pH 4.8. TG and GSSG levels in RCK remained stable at pH 3.0–4.8 (Figure 5). 

**Figure 6:**
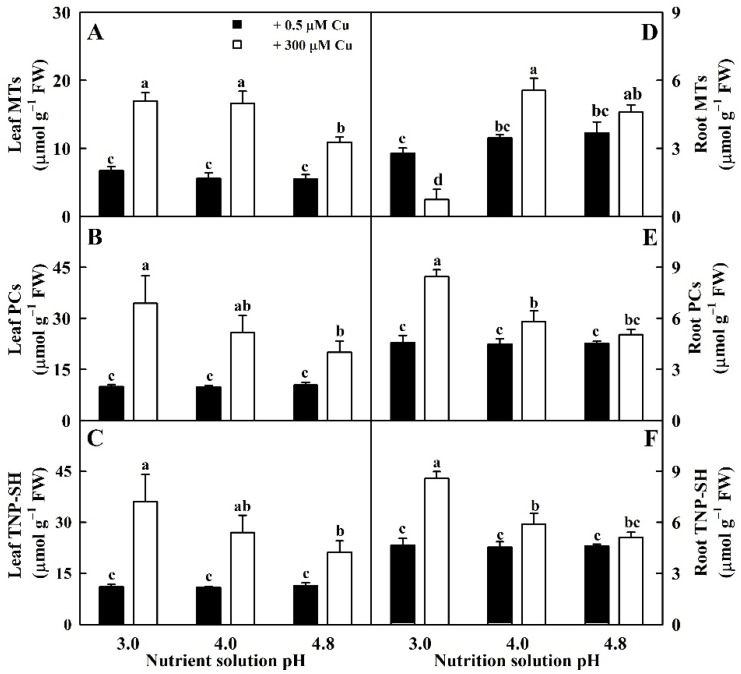
Impacts of Cu–pH interactions on the concentrations of MTs (**A**,**D**), PCs (**B**,**E**), and TNP-SH (**C**,**F**) in leaves and roots. Bars represent means ± SE (*n* = 4). Different letters above the bars indicate a significant difference at *p* < 0.05.

Cu toxicity increased total non-protein thiols (TNP-SH), MTs, and PCs concentrations in leaves more at pH 3.0 than at pH 4.8. MT concentration in roots was decreased, increased, and unaltered by Cu toxicity at pH 3.0, 4.0, and 4.8, respectively. Cu toxicity elevated PC and TNP-SH concentrations in roots at pH 3.0–4.0 but had no effect on them at pH 4.8. Low pH enhanced the levels of TNP-SH and PCs in LCu300 and RCu300 and MTs in LCu300 relative to pH 4.8 but reduced MT levels in RCu300. The three parameters in LCK and RCK remained unchanged at pH 3.0–4.8 (Figure 6). 

### 2.4. Pearson Correlation Coefficient Matrix for All 32 Parameters in Leaves and/or Roots

In leaves, a positive correlation existed between any two parameters of SAPR, HPR, MDA, and MG. SAPR, HPR, MDA, and MG were positively related to SOD, γGCS, γGT, Gly II, TNP-SH, PCs, MTs, ASC, DHA, and TA, respectively—with the few exceptions including the relationships between MG and Gly II, MG and TA, MG and ASC, MG and MTs, MG and PCs, and MG and TNP-SH—and negatively related to CAT, APX, DHAR, MDHAR, GR, GuPX, ATPS, APR, CS, GS, GST, Gly I, TG, GSH, GSSG, GSH/TG, and ASC/TA, respectively—with the few exceptions including the relationships between SAPR and ASC/TA, MG and GuPX, HPR and APR, MG and APR, MG and CS, MG and Gly I, MDA and ASC/TA, SAPR and GSSG, and MDA and GSSG (Table S1). 

In roots, a positive correlation existed between any two parameters of SAPR, HPR, MDA, and MG. SAPR, HPR, MDA, and MG were positively related to GuPX, SiR, PCs, and TNP-SH, respectively, and negatively related to CAT, APX, DHAR, MDHAR, GR, SOD, ATPS, APR, CS, γGCS, GS, GST, Gly I, Gly II, TG, GSH, GSSG, GSH/TG, and ASC/TA, respectively (Table S2).

As shown in Table S3, 21 (HPR, SAPR, MDA, MG, CAT, APX, DHAR, MDHAR, GR, ATPS, APR, CS, GS, GST, Gly I, GSH, TG, GSH/TG, PCs, TNP-SH, and ASC/TA) and 4 (GuPX, SOD, γGCS, and Gly II) parameters in leaves displayed positive and negative relations with the corresponding root parameters, respectively.

### 2.5. PCA Loading Plots

PCA suggested that low pH increased Cu-toxicity-induced-separation of the 64 parameters, and Cu toxicity intensified low-pH-induced-separation of the 64 parameters (Figure S2). 

## 3. Discussion

### 3.1. Impacts of Cu Toxicity and Low pH on ROS and MG Formation and Removal in Leaves and Roots Displayed Obvious Synergisms

**Figure 7:**
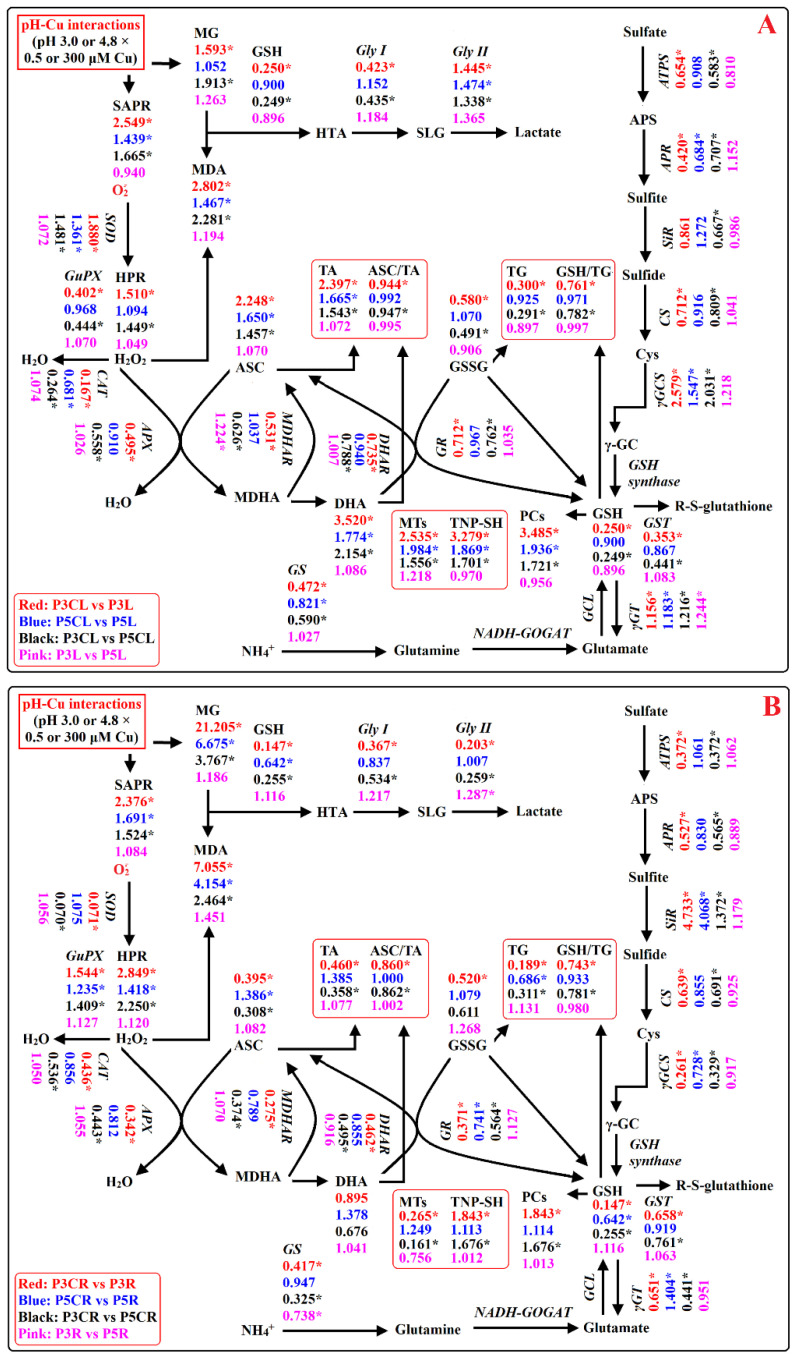
A diagram displaying the effects of Cu–pH interactions on ROS and MG metabolisms in leaves (**A**) and roots (**B**). In this figure, we used italics for enzymes and plain format for metabolites. An asterisk indicates a significant difference between two treatments of a comparative group at *p* < 0.05. An enzyme or metabolite was considered increased (decreased) when it had both a relative change of more (less) than 1 and a *p*-value of < 0.05. P3L, pH 3.0 + 0.5 μM Cu-treated leaves; P3R, pH 3.0 + 0.5 μM Cu-treated roots; P5L, pH 4.8 + 0.5 μM Cu-treated leaves; P5R, pH 4.8 + 0.5 μM Cu-treated roots; P3CL, pH 3.0 + 300 μM Cu-treated leaves; P3CR, pH 3.0 + 300 μM Cu-treated roots; P5CL, pH 4.8 + 300 μM Cu-treated leaves; and P5CR, pH 4.8 + 300 μM Cu-treated roots.

The impacts of Cu toxicity on most parameters involved in ROS and MG production and removal were more pronounced in pH-3.0- than in pH-4.8-treated seedlings. Cu toxicity had no impacts on 2 and 37 out of 64 parameters in pH-3.0- and pH-4.8-treated seedlings, respectively. Low pH impaired the balance between ROS and MG formation and removal, thereby causing oxidative damage in LCu300 and RCu300 but not in LCK and RCK. Indeed, low pH affected 62 out of 64 parameters at 300 μM Cu relative to pH 4.8, but only 4 out of 64 parameters at 0.5 μM Cu (Figure 1, Figure 2, Figure 3, Figure 4, Figure 5, Figure 6 and Figure 7). PCA showed that Cu toxicity aggravated low pH impacts on the 64 parameters and vice versa (Figure S2). Interactive impacts of Cu–pH on 49 out of 64 parameters were significant (Table S4). Thus, Cu toxicity and low pH had synergistic effects on ROS and MG production and removal in leaves and roots. This was supported by the findings that Cu toxicity and low pH had synergistic impacts on seedling growth (Figure S1) and increased pH mitigated aluminum (Al)-toxicity-induced oxidative damage and that Al toxicity aggravated low pH-induced oxidative damage in sweet orange leaves and roots [23].

### 3.2. Elevated pH Ameliorated Oxidative Damage in LCu300 and RCu300

We observed a positive correlation between any two parameters of HPR, SAPR, MDA, and MG (Tables S1 and S2), demonstrating that Cu toxicity triggered ROS and MG generation and overaccumulation, thereby causing oxidative damage in leaves and roots. Cu-toxicity-induced ROS and MG formation and oxidative damage have been obtained in *Citrus* [19,30,31] and *Malus prunifolia* [12] leaves and roots, *Brassica rapa* roots and shoots [32], *Linum usitatissimum* shoots [20], *V. radiata* leaves [21], and *Saccharum officinarum* roots [22].

Our results clearly demonstrated that high pH prevented Cu-toxicity-induced increases in HPR, SAPR, MG, and MDA concentrations in leaves and roots and that Cu toxicity increased HPR, SAPR, MG, and MDA levels more in roots than in leaves. The different responses of the four parameters in leaves and roots to Cu toxicity could be explained by the preferential accumulation of Cu in Cu-toxic roots because Cu fraction in sweet orange roots increased in response to Cu toxicity [33].

ROS can be detoxified by enzymatic and non-enzymatic (ASC, GSH, PCs, MTs, and TNP-SH) scavenging systems. MG removal is primarily catalyzed by glyoxalases using cofactor GSH [24]. Yang et al. [23] reported that elevated-pH-mediated mitigation of oxidative damage caused by Al toxicity in leaves and roots involved the coordinated actions of ROS and MG scavenging systems. In addition to scavenging ROS and MG, GSH, PCs, and MTs can mitigate plant Cu toxicity via the chelation of Cu ions in the cytosol. Cu-PCs and/or Cu-GSH formed can be isolated into vacuoles by ABC transporter [12]. Additionally, GSH can serve as the precursor of PCs biosynthesis. Navarrete et al. [34] demonstrated the involvement of a coordinated and complementary induction of GSH, PCs, and MTs in the detoxification of Cu toxicity in *Ulva compressa*. Mostofa et al. [35] reported that the decreases in Cu uptake and ROS accumulation and the increases in redox state; ASC and PCs concentrations; and GST, DHAR, and CAT activities played a key role, at least partially, in GSH-mediated amelioration of oxidative damage in Cu-toxic rice leaves. 

We found that elevated pH ameliorated Cu-toxicity-induced reductions in the activities of five antioxidant enzymes (APX, CAT, DHAR, MDHAR, and GR); five sulfur-metabolism-related enzymes (ATPS, APR, CS, GS, and GST); Gly I in leaves and roots; GuPX in leaves; SOD, γGCS, γGT, and Gly II in roots; the levels of GSSG, GSH, and TG in leaves and roots; MTs, ASC, and TA in roots; and GSH and ASC redox state in roots and leaves. It ameliorated Cu-toxicity-induced increments in the activities of SOD, γGCS, γGT, and Gly II in leaves; GuPX and SiR in roots; the levels of TNP-SH and PCs in leaves and roots; and DHA, ASC, TA, and MTs in leaves. Cu toxicity did not affect the activities of SiR in pH-3.0- and pH-4.8-treated leaves and the levels of DHA in pH-3.0- and pH-4.8-treated roots (Figure 1, Figure 2, Figure 3, Figure 4, Figure 5 and Figure 6). We observed that MDA was positively related to PCs, TNP-SH, HPR, SAPR, or MG in leaves and roots; SOD, γGCS, γGT, Gly II, TA, ASC, DHA, or MTs in leaves; and GuPX or SiR in roots and negatively related to CAT, APX, DHAR, MDHAR, GR, ATPS, APR, CS, GS, GST, Gly I, GSH + GSSG, GSH, or GSH/TG in leaves and roots; GuPX in leaves; and SOD, γGCS, Gly II, GSSG, or ASC/TA in roots (Tables S1 and S2). It is known that the coordinated actions of glyoxalase and antioxidant detoxification systems play a key role in dealing with the increased generation of ROS and MG [24]. Exogenous application of acetylsalicylic acid and NO alleviated *V. radiata* Cu toxicity by improving the activities of antioxidant enzymes and glyoxalases and decreasing H_2_O_2_, superoxide anion, MG, and MDA accumulation in Cu-toxic leaves [21]. These results suggested that elevated-pH-mediated alleviation of oxidative damage involved enhanced ability to keep the balance between ROS and MG generation and removal via the downregulation of ROS and MG formation and the upregulation of ROS and MG removal systems in leaves and roots.

In plant cells, the concentrations of antioxidant metabolites depend on their biosynthesis, degradation, and utilization. Our results indicated that Cu toxicity greatly reduced GSH concentrations in pH-3.0-treated leaves and roots but only slightly reduced GSH concentration in pH-4.8-treated roots and had no significant effect on GSH concentration in pH-4.8-treated leaves (Figure 5). In leaves, GSH was positively related to GR, ATPS, GS, GST, or Gly I but was negatively related to γGCS, γGT, or PCs (Table S1). Cu-toxicity-induced reduction in GSH concentration in pH-3.0-treated leaves was associated with reduced regeneration due to lessened GR activity and increased utilization for the biosynthesis of PCs. In roots, GSH was positively related to GR, ATPS, APR, CS, γGCS, GST, or Gly I but was negatively related to SiR, γGCS, γGT, or PCs (Table S2). Cu-toxicity-induced reduction in GSH concentration in pH-3.0-treated roots was associated with decreased biosynthesis due to reduced CS and γGCS and regeneration due to reduced GR and also with elevated utilization for the biosynthesis of PCs. Cu-toxicity-induced large increases in MTs in pH-3.0-treated leaves and in PCs and TNP-SH in pH-3.0-treated leaves and roots agreed with the elevated requirement for Cu chelation and sequestration because Cu toxicity greatly elevated Cu accumulation in leaves and roots, especially at low pH [3]. In *Brassica napus*, 100 μM Cu-treated roots suffered from oxidative stress, displaying decreases in TG and GSH concentrations, and GSH redox state, while TG and GSH concentrations in 100 μM Cu-treated leaves displayed a peak at 5 min of Cu treatment and then declined with the extension of Cu treatment time [36]. Cu toxicity increased the concentrations of GSH, GSSG, and PCs in cucumber roots and leaves [26] and in rice leaves [35]. In *U. compressa*, Cu-induced alterations in the concentrations of GSH, PCs, and MTs varied with Cu concentration and duration of exposure to Cu [34]. Taken together, the impacts of Cu on the concentrations of GSH, GSSG, PCs, and MTs and on GSH redox state depended on duration of exposure to Cu, pH, Cu concentration, and plant species and/or tissue.

We found that Cu toxicity enhanced the concentrations of TA, ASC, and DHA in leaves more at pH 3.0 than at pH 4.8 (Figure 5) and that ASC was negatively related to APX, DHAR, and MDHAR, respectively (Table S1). Cu-toxicity-induced increment of ASC levels in leaves might be caused by reduced degradation due to decreased APX activity. In roots, Cu toxicity increased the concentrations of TA, ASC, and DHA in pH-4.0–4.8-treated roots and decreased their concentrations in pH-3.0-treated roots with the exceptions that Cu toxicity did not significantly alter DHA concentration in pH-3.0- and pH-4.8-treated roots (Figure 5). Cu-toxicity-induced reduction in ASC concentration in pH-3.0-treated roots might be caused by reduced regeneration due to decreased DHAR and MDHAR activities (Figure 2). Interestingly, Cu toxicity led to decreased ASC redox state in pH-3.0-treated leaves and roots but not in pH-4.0–4.8 treated leaves and roots (Figure 5). In rice, Cu toxicity led to decreased ASC concentration and redox state and increased APX, DHAR, and MDHAR activities and DHA concentration in leaves [35]. In *V. radiata*, Cu toxicity improved APX activity and lowered ASC concentration in leaves [21]. In wheat, Cu-toxic leaves displayed increases in TA and ASC concentrations and in APX, DHAR, and MDHAR activities [27]. Drążkiewicz et al. [37] examined the operation of ASC-glutathione cycles in leaves of *Arabidopsis* seedlings submitted to 0, 5, 25, 50, and 100 μM Cu for 1, 3, and 7 days. Changes in the activities of APX, MDHAR, and DHAR, and concentrations of ASC and DHA were both time- and dose-dependent. In *Medicago sativa*, APX activity in shoots slightly declined with the increase in Cu supply, while APX activity in roots increased at high Cu concentrations; Cu treatments decreased or did not affect ASC concentration and ASC redox state and increased or did not affect DHA concentration with the exception that 50 mg kg^−1^ Cu increased ASC concentration in shoots; Cu treatments increased or did not alter ASC concentration and ASC redox state and decreased or did not affect DHA concentration in roots [38]. Thus, it appeared that the impacts of Cu on ASC, DHA, and TA concentrations and on ASC redox state depended on duration of Cu treatment, Cu concentration, pH, and plant species and/or tissue. 

Cu toxicity enhanced MG concentration in leaves at pH 3.0, but not at pH 4.0–4.8 (Figure 1). MG was negatively correlated with GSH and displayed an elevated trend with the reduction of Gly I in leaves (Table S1). Cu-toxicity-induced increase in MG concentration in leaves at pH 3.0 could be caused by Cu-toxicity-triggered generation of MG and downregulated ability to scavenge MG due to decreased Gly I activity and GSH levels. In roots, Cu-toxicity-induced increment of MG accumulation elevated with the reduction in pH (Figure 1). MG was negatively correlated with Gly I, Gly II, or GSH in roots (Table S2). Cu-toxicity-induced increase in MG concentration in roots involved upregulated generation and downregulated detoxification of MG due to reduced GSH level and Gly I and Gly II activities. In *V. radiata* leaves, Cu-toxicity-induced accumulation of MG was caused by increased MG formation rather than by decreased capacity to detoxify MG because Cu toxicity led to increased Gly I and Gly II activities and GSH concentration [21]. In rice leaves, Cu toxicity increased GSH and MG concentrations 4 and 7 days after Cu treatment, and their concentrations at 7 days were higher than those at 4 days; Cu toxicity increased Gly I and Gly II activities at 4 days and decreased (did not alter) Gly I (Gly II) activity at 7 days. Cu-toxicity-induced accumulation of MG in rice leaves was due to increased MG formation and decreased MG removal at 7 days and due to increased MG generation at 4 days [39]. 

### 3.3. Some Differences Existed in the Impacts of Cu–pH Interactions on ROS and MG Metabolisms between Leaves and Roots

As shown in Table S3, 21 (4) parameters in leaves were positively (negatively) related to the corresponding root parameters, and there was no significant correlation between seven parameters in leaves and the corresponding parameters in roots, implying that some differences existed in the Cu–pH-interaction-induced changes in ROS and MG metabolisms between leaves and roots. For example, Cu toxicity elevated HPR, SAPR, MG, MDA, TG, and GSH concentrations more in roots than in leaves (Figure 1 and Figure 5). Cu toxicity elevated the activities of GuPX in roots and of SOD, γGCS, and Gly II in leaves as well as the concentration of MTs in leaves but reduced or did not significantly influence the activities of GuPX in leaves; of SOD, γGCS, and Gly II in roots; or of the concentration of MTs in roots. TA, ASC, and DHA concentrations were increased by Cu toxicity in pH-3.0–4.8-treated leaves and in pH-4.0–4.8-treated roots but decreased or were unaffected by Cu toxicity in pH-3.0-treated roots. Low pH reduced the activities of GuPX in RCu300 and SOD, γGCS, γGT, and Gly II in LCu300 and the concentrations of TA, ASC, and MTs in LCu300, but it decreased the activities of GuPX in LCu300 and SOD, γGCS, γGT, and Gly II in RCu300 as well as the concentrations of TA, ASC, and MTs in RCu300 (Figure 2, Figure 3, Figure 4, Figure 5 and Figure 6). 

## 4. Materials and Methods

### 4.1. Plant Materials

The culture and Cu–pH treatments of sweet orange (*Citrus sinensis* (L.) Osbeck cv. Xuegan) seedlings referred to Cai et al. [3]. Six-week-old uniform seedlings were transplanted to 6 L pots filled with sand (two plants per pot) and then cultured in the greenhouse under natural conditions at Fujian Agriculture and Forestry University, Fuzhou with annual average sunlight, temperature, and relative humidity of ~1600 h, 76%, and 20 °C, respectively [40]. Six weeks after transplantation, each pot was supplemented with freshly prepared nutrient solution six times weekly with 0.5 (control or non-Cu toxicity) or 300 (Cu toxicity) μM CuCl_2_ × pH 4.8, 4.0 or 3.0 (adjusted by 1 M HCl) until dripping (~ 500 mL). These values were chosen after referring to previous reports [2,3]. We found that 300 μM Cu led to a significant—but not too great—decrease in *C. sinensis* seedling growth at pH 3.0, and it had almost no impact on seedling growth at pH 4.8 [2,3]. Without Cu-toxicity, pH 3.0 slightly inhibited seedling growth, pH 4.0 had almost no impact on seedling growth, and seedling growth and many physiological parameters displayed their maximums at pH 5.0 [41]. According to our observations, Cu precipitation formed easily in nutrient solution with pH ≥ 5. A total of 40 seedlings (20 pots) per treatment were arranged in a completely randomized design. Seventeen weeks after treatments, the recently fully explained leaves and ~5 mm in length of white root tips were taken, immediately placed into liquid nitrogen, and stored in a −80 °C freezer until use except for SAPR and HPR.

### 4.2. Measurements of SAPR, HPR, MG, and MDA in Leaves and Roots

SAPR and HPR were measured by the reduction in nitroblue tetrazolium (NBT) and oxidation of guaiacol, respectively [42]. MG was quantified spectrophotometrically based on the N-acetyl-L-cysteine reaction after being extracted with 5% (*w*/*v*) HClO_4_ [43]. MDA was estimated with the improved assay of thiobarbituric-acid-reactive substances after being extracted with 80% (*v*/*v*) ethanol [44].

### 4.3. Assay of Antioxidant Metabolites in Leaves and Roots

MTs were assayed spectrophotometrically using 5,5-dithiobis-2-nitrobenzoic acid (DTNB) after being extracted with buffer containing 20 mM TRIS-HCl (pH 8.6), 0.5 M sucrose, and 0.01% β-mercaptoethanol [45]. TNP-SH and GSH were determined spectrophotometrically using DTNB after being extracted with 5% (*w*/*v*) trichloroacetic acid (TCA). PCs were calculated as: PCs = TNP-SH - GSH [46]. GSSG and TG were measured utilizing GR and DTNB after being extracted with 5% (*w*/*v*) TCA; and TA and ASC were quantified using ASC oxidase after being extracted with 6% (*v*/*v*) HClO_4_ [42]. 

### 4.4. Assay of Enzyme Activities in Leaves and Roots

APX, CAT, GuPX, DHAR, MDHAR, GR, SOD, and GST were extracted according to Guo et al. [47]. Briefly, five frozen leaf discs (6 mm in diameter) or 60 mg frozen root tips were extracted using 2 mL of 50 mM KH_2_PO_4_-KOH (pH 7.5) containing 1 mM ethylene diamine tetraacetic acid (EDTA), 0.5% Triton X-100, and 5% insoluble polyvinylpolypyrrolidone (PVPP). The extract was then centrifuged at 13,000× *g* for 10 min at 4 °C, and the supernatant was used immediately for enzyme activity assay. APX activity was assayed by following the decrease in absorbance at 290 nm. The reaction mixture contained 50 mM HEPES-KOH (pH 7.6), 0.1 mM EDTA, 0.2 mM H_2_O_2_, 0.5 mM ASC, and 50 μL enzyme extract. The reaction was initiated by adding H_2_O_2_ [48]. CAT activity was assayed at 240 nm in 1 mL of reaction mixture containing 100 mM potassium phosphate buffer (pH 6.0), 10 μL 10% (*w*/*v*) H_2_O_2_ and 10 μL enzyme extract. The reaction was initiated by adding H_2_O_2_ [48]. GuPX activity was measured at 470 nm in 1 mL of reaction mixture containing 100 mM potassium phosphate (pH 6.0), 5 μL 10% (*w*/*v*) H_2_O_2_, 16 mM guaiacol, and 10 μL enzyme extract. The reaction was initiated by adding enzyme extract [49]. DHAR activity was assayed at 265 nm in 1 mL of reaction mixture containing 100 mM HEPES-KOH (pH 7.0), 0.1 mM EDTA, 2.5 mM GSH, 0.2 mM DHA, and 100 μL enzyme extract. The reaction was started by adding DHA [48]. MDHAR activity was measured at 340 nm in 1 mL of reaction mixture containing 50 mM HEPES-KOH (pH 7.6), 2.5 mM ASC, 0.1 mM NADH, 0.25 U of ASC oxidase, and 100 μL enzyme extract. The reaction was started by adding ASC oxidase [48]. GR activity was assayed at 340 nm in 1 mL of reaction mixture containing 100 mM TRIS-HCl (pH 8.0), 1 mM GSSG, 1 mM EDTA, 0.2 mM NADPH, and 100 μL enzyme extract. The reaction was started by adding NADPH. SOD activity was assayed at 560 nm using a photochemical assay system consisting of NBT, riboflavin, methionine and enzyme extract. One U of SOD activity is defined as the amount to produce a 50% inhibition of NBT photoreduction [50]. GST activity was assayed in 340 nm in 1 mL of reaction mixture containing 100 mM TRIS-HCl (pH 6.5), 1.5 mM GSH, 1 mM 1-chloro-2,4-dinitrobenzene (CDNB), and 100 μL enzyme extract. The reaction was started by the addition of CDNB [51].

ATPS, CS, APR, SiR, γGT, γGCS, Gly I, and Gly II were extracted according to Cai et al. [52]. Briefly, six frozen leaf discs (6 mm in diameter) or ~100 mg frozen root tips were ground with a precooled mortar and pestle in 2 mL of ice-cold extraction buffer containing 100 mM TRIS-HCl (pH 8.0), 10 mM EDTA, 2 mM dithiothreitol (DTT), and 4% (*w*/*v*) insoluble PVPP. The extract was centrifuged at 13,000× *g* for 10 min at 4 °C. The resultant supernatant was used immediately for enzyme activity assay. ATPS activity was measured according to Guo et al. [47]. An amount of 100 μL of enzyme extract was incubated for 15 min at 37 °C with 80 mM TRIS-HCl (pH 8.0), 5 mM Na_2_MoO_4_, 2 mM Na_2_ATP, 7 mM MgCl_2_, and 0.032 U mL^−1^ of sulfate-free inorganic pyrophosphatase at a total volume of 0.6 mL. The reaction was initiated by the addition of enzyme extract and terminated by adding 2 mL of 20% (*w*/*v*) TCA, and the phosphate yield in the reaction was assayed according to Ames [53]. The blank contained the same reaction mixture and enzyme extract except that Na_2_MoO_4_ was absent. CS activity was measured as described by Warrilow and Hawkesford [54]. The reaction mixture (0.8 mL) contained 0.2 M TRIS-HCl (pH 7.5), 10 mM DTT, 3 mM Na_2_S, and 20 μL extract. This reaction was initiated by adding 5 mM O-acetyl-L-serine (OAS). After 10 min incubation at 25 °C, the reaction was stopped by the addition of 0.2 mL of 1.5 M TCA. The amount of cysteine synthesized was measured using the ninhydrin method. APR activity was determined according to Trüper and Rogers [55] with some modifications. Briefly, 1 mL of reaction mixture for ferricyanide-coupled assay contained 50 mM TRIS-HCl (pH 8.0), 0.5 mM K_3_Fe(CN)_6_, 8 mM EDTA, 0.4 mM adenosine 5′-monophosphate (AMP), 4 mM Na_2_SO_3_, and 100 μL enzyme extract. Absorbance was measured at 420 nm, 25 °C against a blank containing the reaction mixture without enzyme extract. SiR were assayed in 1 mL reaction mixture containing 10 mM TRIS-HCl (pH 7.5), 0.1 mM EDTA, 0.5 mM Na_2_SO_3_, 0.2 mM NADPH, and 100 μL enzyme extract. The reaction was started by the addition of enzyme extract. Absorbance was measured at 340 nm, 25 °C against a blank containing the reaction mixture without enzyme extract [52]. γGT was assayed as described previously [52]. An amount of 1 mL of reaction mixture contained 100 mM TRIS-HCl (pH 8.0), 20 mM glycylglycine (Gly-Gly), 2.5 mM L-γ-glutamyl-p-nitroanilide, and 100 μL enzyme extract. After 30 min incubation at 30 °C, the reaction was stopped by the addition of 1 mL of 25% (*w*/*v*) TCA. The resultant p-nitroaniline (ε = 1.74 mM^−1^ cm^−1^) was measured at 405 nm. γGCS was assayed in 1 mL of reaction buffer containing 100 mM TRIS-HCl (pH 8.0), 20 mM MgCl_2_, 150 mM KCl, 2 mM EDTA, 2 mM phosphoenolpyruvate (PEP), 5 mM ATP, 10 mM glutamate, 10 mM α-aminobutyrate, 0.2 mM NADH, 7 U of pyruvate kinase (PK), 10 U of lactate dehydrogenase (LDH), and 100 μL enzyme extract. The reaction was started by the addition of enzyme extract. Absorbance was measured at 340 nm, 25 °C against a blank containing the reaction buffer without enzyme extract [52]. Gly I activity was assayed in 1 mL of reaction mixture containing 100 mM potassium phosphate buffer (pH 7.0), 15 mM MgSO_4_, 1.7 mM GSH, 3.5 mM MG, and 100 μL enzyme extract. The reaction was started by the addition of MG; the increase in absorbance was measured at 240 nm, 25 °C against a blank containing the reaction mixture without enzyme extract [56]. Gly II activity was determined by monitoring the formation of GSH at 412 nm, 25 °C. The reaction mixture contained 100 mM TRIS-HCl (pH 7.2), 0.2 mM DTNB, 1 mM S-D-lactoylglutathione (SLG), and 100 μL enzyme extract [56]. 

GS was extracted and assayed as described previously [57]; six frozen leaf discs (6 mm in diameter) or ~100 mg frozen root tips were ground with a precooled mortar and pestle in 1 mL of ice-cold extraction buffer containing 100 mM TRIS-HCl (pH 7.6), 1 mM MgCl_2_, 1 mM-EDTA, and 5 mM β-mercaptoethanol and centrifuged at 16,000× *g* for 30 min at 4 °C. Then, 0.3 mL of supernatant was incubated with 1.4 mL of reaction mixture containing 80 mM TRIS-HCl (pH 8.0), 40 mM MgCl_2_, 40 mM monosodium glutamate, and 16 mM hydroxylamine hydrochloride and 0.6 mL of 8 mM ATP in a water bath at 30 °C for 30 min. Blanks (controls) without hydroxylamine hydrochloride were carried through for all samples. The reaction was terminated by adding 1 mL of ingrain agent containing 2% TCA (*w*/*v*), 3.5% FeCl_3_ (*w*/*v*) and 1.8% HCl (*v*/*v*), held at room temperature for 30 min, and centrifuged at 10,000× *g* for 5 min. Finally, absorbance of the supernatant was measured at 540 nm.

### 4.5. Data Analysis

Data were analyzed using two-way ANOVA (two (Cu levels) × three (pH levels)) and then a least significant difference (LSD) post hoc test at *p* < 0.05 using DPS 7.05 (Hangzhou RuiFeng Information Technology Co., Ltd., Hangzhou, China).

## 5. Conclusions

Elevated-pH-mediated mitigation of Cu-toxicity-induced oxidative damage in leaves and roots involved enhanced ability to maintain the balance between ROS and MG generation and removal through the downregulation of ROS and MG formation and the coordinated actions of ROS and MG detoxification systems. Low pH impaired the balance between ROS and MG generation and scavenging, thereby causing oxidative damage in LCu300 and RCu300 but not in LCK or RCK. Cu toxicity and low pH had obvious synergistic impacts on ROS and MG production and removal in leaves and roots. There were some differences between leaves and roots in Cu–pH-interaction-induced changes in ROS and MG metabolisms. To conclude, our findings provided some novel evidence for the contribution of ROS and MG formation and removal in high-pH-mediated mitigation of plant Cu toxicity.

## Data Availability

Data are archived in L.-S. Chen’s lab and are available upon request.

## Supplementary Materials

The following supporting information can be downloaded at: https://www.mdpi.com/article/10.3390/ijms232213896/s1, Figure S1: Effects of pH–copper interactions on growth of *Citrus sinensis* seedlings; Figure S2: Principal component analysis (PCA) loading plots for 64 biochemical parameters in pH-3.0- (A), pH-4.0- (B) and pH-4.8 (C)-treated *Citrus sinensis* leaves and roots at different Cu (0.5 and 300 μM) levels and in 300 (D) and 0.5 (E) μM Cu-treated *C. sinensis* leaves and roots at different pH (3.0, 4.0, and 4.8) levels; Table S1: Pearson correlation coefficient matrix for the mean values of 32 biochemical parameters in *Citrus sinensis* leaves; Table S2: Pearson correlation coefficient matrix for the mean values of 32 biochemical parameters in *Citrus sinensis* roots; Table S3: Pearson correlation coefficient matrix between *Citrus sinensis* leaves (first row) and roots (first column) for the mean values of 32 biochemical parameters; Table S4: Two-way ANOVA for all biochemical parameters in leaves and roots.

## References

[1] Ren Q.-Q., Huang Z.-R., Huang W.-L., Huang W.-T., Chen H.-H., Yang L.-T., Chen L.-S. (2022). Physiological and molecular adaptations of *Citrus grandis* roots to long-term copper excess revealed by physiology, metabolome and transcriptome. Environ. Exp. Bot..

[2] Zhang J., Chen X.-F., Huang W.-T., Chen H.-H., Lai N.-W., Yang L.-T., Huang Z.-R., Guo J.-X., Ye X., Chen L.-S. (2022). Mechanisms for increased pH-mediated amelioration of copper toxicity in *Citrus sinensis* leaves using physiology, transcriptomics and metabolomics. Environ. Exp. Bot..

[3] Cai L.-Y., Zhang J., Ren Q.-Q., Lai Y.-H., Peng M.-Y., Deng C.-L., Ye X., Yang L.-T., Huang Z.-R., Chen L.-S. (2021). Increased pH-mediated alleviation of copper-toxicity and growth response function in *Citrus sinensis* seedlings. Sci. Hortic..

[4] Mir A.R., Pichtel J., Hayat S. (2021). Copper: Uptake, toxicity and tolerance in plants and management of Cu-contaminated soil. BioMetals.

[5] Wu F., Huang H., Peng M., Lai Y., Ren Q., Zhang J., Huang Z., Yang L., Rensing C., Chen L. (2021). Adaptive responses of *Citrus grandis* leaves to copper toxicity revealed by RNA-Seq and physiology. Int. J. Mol. Sci..

[6] Huang W.-L., Wu F.-L., Huang H.-Y., Huang W.-T., Deng C.-L., Yang L.-T., Huang Z.-R., Chen L.-S. (2020). Excess copper-induced alterations of protein profiles and related physiological parameters in *Citrus* leaves. Plants.

[7] Li Q., Chen H.-H., Qi Y.-P., Ye X., Yang L.-T., Huang Z.-R., Chen L.-S. (2019). Excess copper effects on growth, uptake of water and nutrients, carbohydrates, and PSII photochemistry revealed by OJIP transients in *Citrus* seedlings. Environ. Sci. Pollut. Res..

[8] Alengebawy A., Abdelkhalek S.T., Qureshi S.R., Wang M.Q. (2021). Heavy metals and pesticides toxicity in agricultural soil and plants: Ecological risks and human health implications. Toxics.

[9] Schmitt O., Andriolo J.L., Silva I.C.B., Tiecher T.L., Chassot T., Tarouco C.P., Lourenzi C.R., Nicoloso F.T., Marchezan C., Casagrande C.R. (2022). Physiological responses of beet and cabbage plants exposed to copper and their potential insertion in human food chain. Environ. Sci. Pollut. Res..

[10] Adrees M., Ali S., Rizwan M., Ibrahim M., Abbas F., Farid M., Ziaurrehman M., Irshad M.K., Bharwana S.A. (2015). The effect of excess copper on growth and physiology of important food crops: A review. Environ. Sci. Pollut. Res..

[11] Yuan M., Li Y., Zhang C., Wang J., Li S., Fu X., Ling L., Cao L., Peng L. (2018). Review of research on copper stress in *Citrus*. J. Fruit Sci..

[12] Wan H., Du J., He J., Lyu D., Li H. (2019). Copper accumulation, subcellular partitioning and physiological and molecular responses in relation to different copper tolerance in apple rootstocks. Tree Physiol..

[13] Li Y., Han M.-Q., Lin F., Ten Y., Lin J., Zhu D.-H., Guo P., Weng Y.-B., Chen L.-S. (2015). Soil chemical properties, ‘Guanximiyou’ pummelo leaf mineral nutrient status and fruit quality in the southern region of Fujian province. China. J. Soil Sci. Plant Nutr..

[14] Fan J.H., He Z.L., Ma L.Q., Stoffella P.J. (2011). Accumulation and availability of copper in Citrus grove soils as affected by fungicide application. J. Soil Sediment.

[15] Alva A.K., Huang B., Prakash O., Paramasivam S. (1999). Effects of copper rates and soil pH on growth and nutrient uptake by *Citrus* seedlings. J. Plant Nutr..

[16] Trentin E., Cesco S., Pii Y., Valentinuzzi F., Celletti S., Feil S.B., Zuluaga M.Y.A., Ferreira P.A.A., Ricachenevsky F.K., Stefanello L.O. (2022). Plant species and pH dependent responses to copper toxicity. Environ. Exp. Bot..

[17] Ambrosini V.G., Rosa D.J., Basso A., Borghezan M., Pescador R., Miotto A., George de Melo W.B., de Sousa Soares C.R.F., Comin J.J., Brunetto G. (2017). Liming as an ameliorator of copper toxicity in black oat (*Avena strigosa* Schreb.). J. Plant Nutr..

[18] Chen H.-H., Chen X.-F., Zheng Z.-C., Huang W.-L., Guo J., Yang L.-T., Chen L.-S. (2022). Characterization of copper-induced-release of exudates by *Citrus sinensis* roots and their possible roles in copper-tolerance. Chemosphere.

[19] Giannakoula A., Therios I., Chatzissavvidis C. (2021). Effect of lead and copper on photosynthetic apparatus in *Citrus* (*Citrus aurantium* L.) plants. The role of antioxidants in oxidative damage as a response to heavy metal stress. Plants.

[20] El-Beltagi H.S., Sofy M.R., Aldaej M.I., Mohamed H.I. (2020). Silicon alleviates copper toxicity in flax plants by up-regulating antioxidant defense and secondary metabolites and decreasing oxidative damage. Sustainability.

[21] Abdulmajeed A.M., Alnusairi M.H.A., Almushhin A., Hasan M.D., Soliman M.H. (2021). Alleviation of copper phytotoxicity by acetylsalicylic acid and nitric oxide application in mung bean involves the up-regulation of antioxidants, osmolytes and glyoxalase system. J. Plant Interact..

[22] Zeng Q.Y., Ling Q.P., Wu J.Y., Yang Z.D., Liu R., Qi Y.W. (2019). Excess copper-induced changes in antioxidative enzyme activity, mineral nutrient uptake and translocation in sugarcane seedlings. Bull. Environ. Contam. Toxicol..

[23] Yang T.-Y., Huang W.-T., Zhang J., Yang L.-T., Wu B.-S., Lai N.-W., Chen L.-S. (2021). Raised pH conferred the ability to maintain a balance between production and detoxification of reactive oxygen species and methylglyoxal in aluminum-toxic *Citrus sinensis* leaves and roots. Environ. Pollut..

[24] Hasanuzzaman M., Nahar K., Hossain M.S., Mahmud J.A., Rahman A., Inafuku M., Oku H., Fujita M. (2017). Coordinated actions of glyoxalase and antioxidant defense systems in conferring abiotic stress tolerance in plants. Int. J. Mol. Sci..

[25] Tahjib-Ul-Arif M., Sohag A.A.M., Mostofa M.G., Polash M.A.S., Mahamud A.G.M.S.U., Afrin S., Hossain M.A., Hossain M.A., Murata Y., Tran L.P. (2021). Comparative effects of ascobin and glutathione on copper homeostasis and oxidative stress metabolism in mitigation of copper toxicity in rice. Plant Biol..

[26] Cao Y.Y., Qi C.D., Li S., Wang Z., Wang X., Wang J., Guo Y.D. (2019). Melatonin alleviates copper toxicity via improving copper sequestration and ROS scavengingin cucumber. Plant Cell Physiol..

[27] Shan C.J., Dai H.P., Sun Y.F. (2012). Hydrogen sulfide protects wheat seedlings against copper stress by regulating the ascorbate and glutathione metabolism in leaves. Aust. J. Crop Sci..

[28] Yuan X.D., Xue N.D., Han Z.G. (2021). A meta-analysis of heavy metals pollution in farmland and urban soils in China over the past 20 years. J. Environ. Sci..

[29] Zhang S.W., Yang W.H., Muneer M.A., Ji Z.J., Tong L., Zhang X., Li X.X., Wang W.Q., Zhang F.S., Wu L.Q. (2021). Integrated use of lime with Mg fertilizer significantly improves the pomelo yield, quality, economic returns and soil physicochemical properties under acidic soil of southern China. Sci. Hortic..

[30] Hippler F.W.R., Cipriano D.O., Boaretto R.M., Quaggio J.A., Gaziola S.A., Azevedo R.A., Mattos-Jr D. (2016). *Citrus* rootstocks regulate the nutritional status and antioxidant system of trees under copper stress. Environ. Exp. Bot..

[31] Hippler F.W.R., Petená G., Boaretto R.M., Quaggio J.A., Azevedo R.A., Mattos-Jr D. (2018). Mechanisms of copper stress alleviation in *Citrus* trees after metal uptake by leaves or roots. Environ. Sci. Pollut. Res..

[32] Chung I.M., Rekha K., Venkidasamy B., Thiruvengadam M. (2019). Effect of copper oxide nanoparticles on the physiology, bioactive molecules, and transcriptional changes in *Brassica rapa* ssp. *rapa* seedlings. Water Air Soil Pollut..

[33] Chen X.-F., Hua D., Zheng Z.-C., Zhang J., Huang W.-T., Chen H.-H., Huang Z.-R., Yang L.-T., Ye X., Chen L.-S. (2022). Boron-mediated amelioration of copper-toxicity in sweet orange [*Citrus sinensis* (L.) Osbeck cv. Xuegan] seedlings involved reduced damage to roots and improved nutrition and water status. Ecotoxicol. Environ. Saf..

[34] Navarrete A., González A., Gómez M., Contreras R.A., Díaz P., Lobos G., Brown M.T., Sáez C.A., Moenne A. (2019). Copper excess detoxification is mediated by a coordinated and complementary induction of glutathione, phytochelatins and metallothioneins in the green seaweed *Ulva compressa*. Plant Physiol. Biochem..

[35] Mostofa M.G., Seraj Z.I., Fujita M. (2014). Exogenous sodium nitroprusside and glutathione alleviate copper toxicity by reducing copper uptake and oxidative damage in rice (*Oryza sativa* L.) seedlings. Protoplasma.

[36] Russo M., Sgherri C., Izzo R., Navari-Izzo F. (2008). *Brassica napus* subjected to copper excess: Phospholipases C and D and glutathione system in signalling. Environ. Exp. Bot..

[37] Drążkiewicz M., Skórzyńska-Polit E., Krupa Z. (2003). Response of the ascorbate–glutathione cycle to excess copper in *Arabidopsis thaliana* (L.). Plant Sci..

[38] Chen J., Liu Y.Q., Yan X.W., Wei G.H., Zhang J.H., Fang L.C. (2018). Rhizobium inoculation enhances copper tolerance by affecting copper uptake and regulating the ascorbate-glutathione cycle and phytochelatin biosynthesis-related gene expression in *Medicago sativa* seedlings. Ecotoxicol. Environ. Saf..

[39] Mostofa M.G., Hossain M.A., Fujita M., Tran L.S.P. (2015). Physiological and biochemical mechanisms associated with trehalose-induced copper-stress tolerance in rice. Sci. Rep..

[40] Yang T.-Y., Cai L.-Y., Qi Y.-P., Yang L.-T., Lai N.-W., Chen L.-S. (2019). Increasing nutrient solution pH alleviated aluminum-induced inhibition of growth and impairment of photosynthetic electron transport chain in *Citrus* sinensis seedlings. Biomed. Res. Int..

[41] Long A., Zhang J., Yang L.-T., Ye X., Lai N.-W., Tan L.-L., Lin D., Chen L.-S. (2017). Effects of low pH on photosynthesis, related physiological parameters, and nutrient profiles of *Citrus*. Front. Plant Sci..

[42] Chen L.-S., Qi Y.-P., Liu X.-H. (2005). Effects of aluminum on light energy utilization and photoprotective systems in *Citrus* leaves. Ann. Bot..

[43] Wild R., Ooi L., Srikanth V., Münch G. (2012). A quick, convenient and economical method for the reliable determination of methylglyoxal in millimolar concentrations: The N-acetyl-L-cysteine assay. Anal. Bioanal. Chem..

[44] Hodges D.M., DeLong J.M., Forney C.F., Prange R.K. (1999). Improving the thiobarbituric acid-reactive-substances assay for estimating lipid peroxidation in plant tissues containing anthocyanin and other interfering compounds. Planta.

[45] Malik J.A., Goel S., Kaur N., Sharma S., Singh I., Nayyar H. (2012). Selenium antagonises the toxic effects of arsenic on mungbean (*Phaseolus aureus* Roxb.) plants by restricting its uptake and enhancing the antioxidative and detoxification mechanisms. Environ. Exp. Bot..

[46] Garg N., Kaur H. (2013). Response of antioxidant enzymes, phytochelatins and glutathione production towards Cd and Zn stresses in *Cajanus cajan* (L.) Millsp. genotypes colonized by arbuscular mycorrhizal fungi. J. Agron. Crop Sci..

[47] Guo P., Qi Y.-P., Cai Y.-T., Yang T.-Y., Yang L.-T., Huang Z.-R., Chen L.-S. (2018). Aluminum effects on photosynthesis, reactive oxygen species and methylglyoxal detoxification in two *Citrus* species differing in aluminum tolerance. Tree Physiol..

[48] Chen L.-S., Cheng L. (2003). 2003. Both xanthophyll cycle-dependent thermal dissipation and the antioxidant system are up-regulated in grape (*Vitis labrusca* L. cv. Concord) leaves in responses to N limitation. J. Exp. Bot..

[49] Chen L.-S., Li P., Cheng L. (2008). Effects of high temperature coupled with high light on the balance between photooxidation and photoprotection in the sun-exposed peel of apple. Planta.

[50] Giannopolitis C.N., Ries S.K. (1977). Superoxide dismutases I. Occurrence in higher plants. Plant Physiol..

[51] Fujita M., Hossain M.Z. (2003). Modulation of pumpkin glutathione S-transferases by aldehydes and related compounds. Plant Cell Physiol..

[52] Cai Y.-T., Zhang H., Qi Y.-P., Ye X., Huang Z.-R., Guo J.-X., Chen L.-S., Yang L.-T. (2019). Responses of reactive oxygen species and methylglyoxal metabolisms to magnesium-deficiency differ greatly among the roots, upper and lower leaves of *Citrus sinensis*. BMC Plant Biol..

[53] Ames B.N. (1966). Assay of inorganic phosphate, total phosphate and phosphatase. Method Enzymol..

[54] Warrilow A.G.S., Hawkesford M.J. (2000). Cysteine synthase (O-acetylserine (thiol) lyase) substrate specificities classify the mitochondrial isoform as a cyanoalanine synthase. J. Exp. Bot..

[55] Trüper H.G., Rogers L.A. (1971). Purification and properties of adenylyl sulfate reductase from the phototrophic sulfur bacterium, *Thiocapsa roseopersicina*. J. Bacteriol..

[56] Hasanuzzaman M., Hossain M.A., Fujita M. (2011). Nitric oxide modulates antioxidant defense and the methylglyoxal detoxification system and reduces salinity-induced damage of wheat seedlings. Plant Biotechnol. Rep..

[57] Chen H., Jia Y., Xu H., Wang Y., Zhou Y., Huang Z., Ynag L., Li Y., Chen L.-S., Guo J. (2020). Ammonium nutrition inhibits plant growth and nitrogen uptake in *Citrus* seedlings. Sci. Hortic..

