# Supplementary material for: High pH Alleviated Sweet Orange (Citrus sinensis) Copper Toxicity by Enhancing the Capacity to Maintain a Balance between Formation and Removal of Reactive Oxygen Species and Methylglyoxal in Leaves and Roots
Source: Int J Mol Sci. 2022 Nov 11;23(22):13896. doi: 10.3390/ijms232213896 (PMC9698688; doi:10.3390/ijms232213896)
Supplement: Supplementary file 1 [file ijms-23-13896-s001.zip › 2022ZhangIJMS Figures S1-S2 and Table S4.pdf]

Figures S1-S2 and Table S4

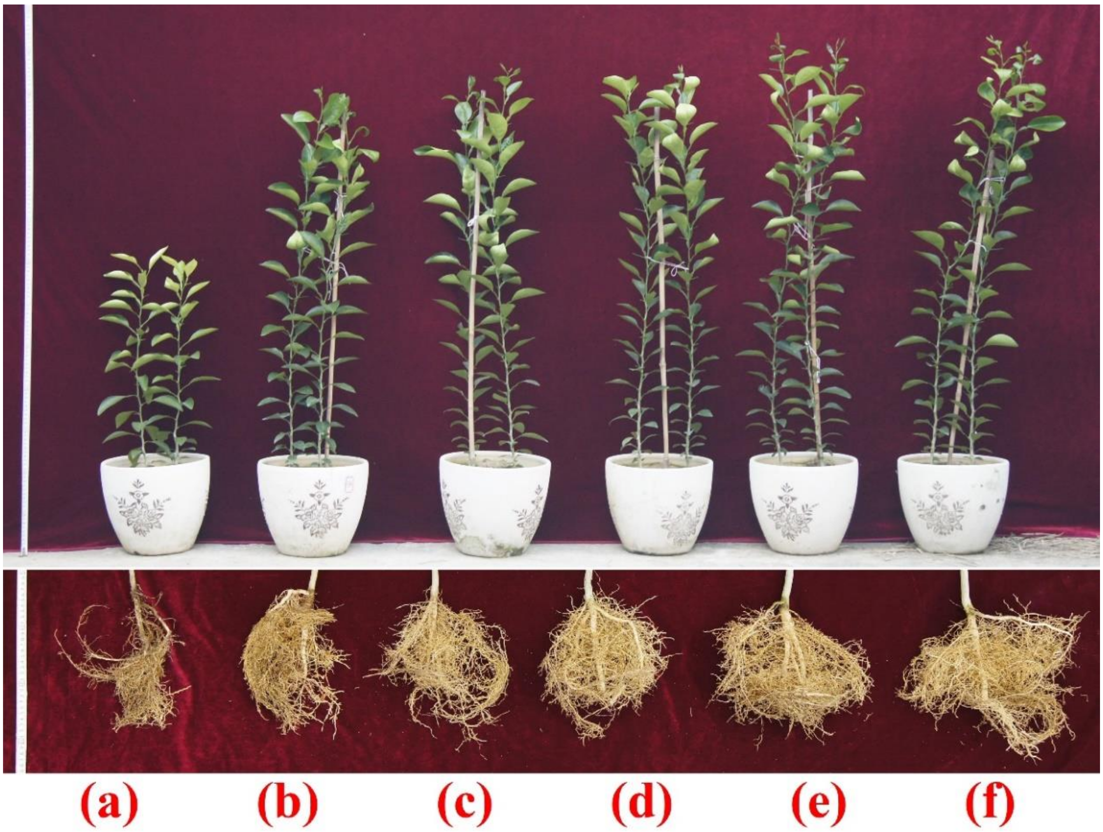

Figure S1: Effects of pH-copper interactions on growth of *Citrus sinensis* seedlings. (a), pH 3.0 + 300 μM Cu; (b), pH 4.0 + 300 μM Cu; (c), pH 4.8 + 300 μM Cu; (d), pH 3.0 + 0.5 μM Cu; (e), pH 4.0 + 0.5 μM Cu; and (f), pH 4.8 + 0.5 μM Cu.

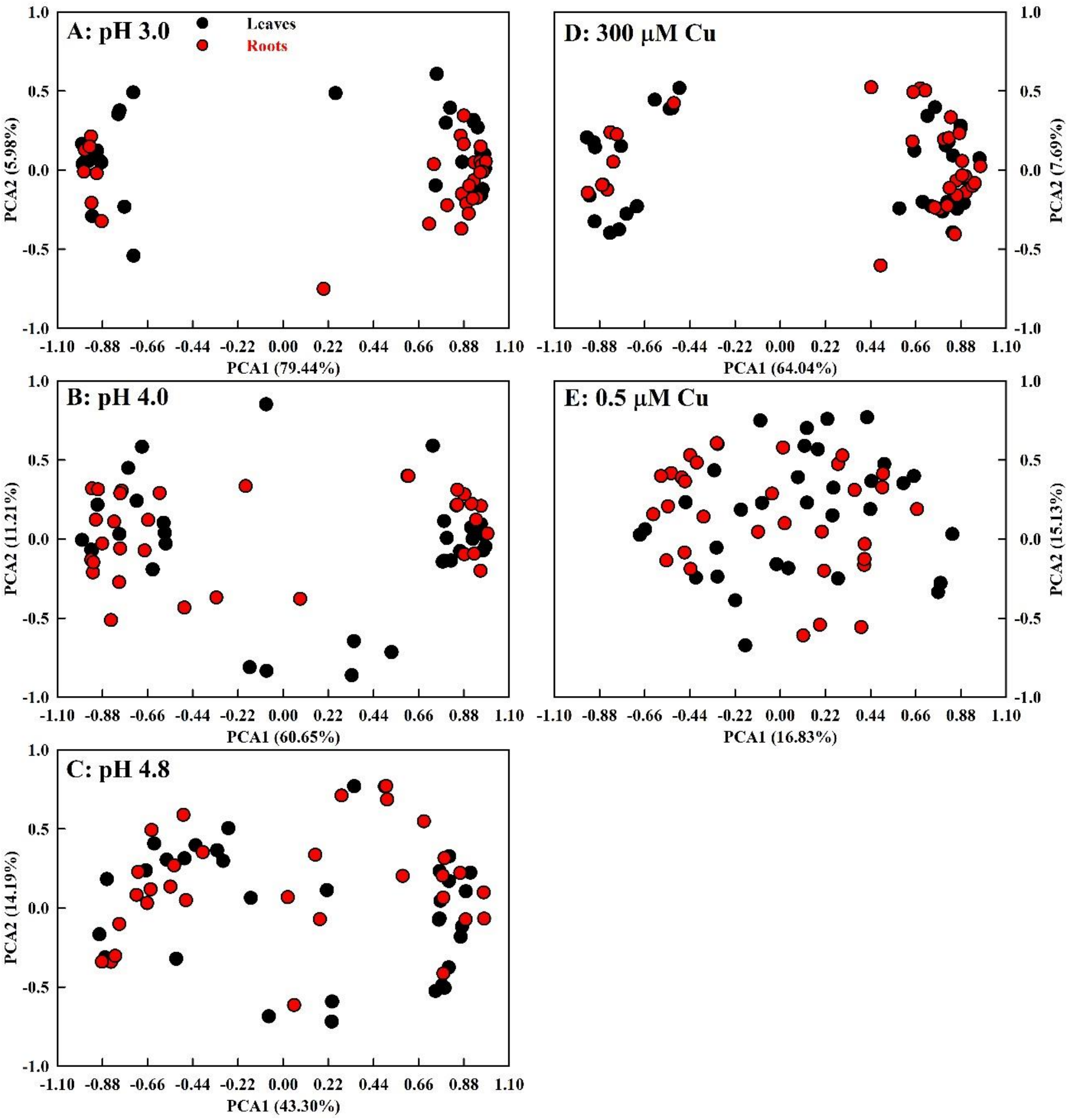

Figure S2: Principal component analysis (PCA) loading plots for 64 biochemical parameters in pH-3.0- (**A**), pH-4.0- (**B**) and pH-4.8- (**C**) treated *Citrus sinensis* leaves and roots at different Cu (0.5 and 300 μM) levels, and in 300 (**D**) and 0.5 (**E**) μM Cu-treated *Citrus sinensis* leaves and roots at different pH (3.0, 4.0 and 4.8) levels.

Table S4: Two-way ANOVA for all biochemical parameters in leaves and roots

Parameters	Tissue	Cu		pH		Cu × pH	
		F	*p*	F	*p*	F	*p*
HPR	Leaves	55.368	0.0001	22.199	0.0001	13.936	0.0002
	Roots	52.191	0.0001	11.420	0.0006	8.722	0.0022
SAPR	Leaves	247.861	0.0001	21.869	0.0001	29.092	0.0001
	Roots	103.300	0.0001	7.832	0.0036	5.304	0.0155
MDA	Leaves	121.648	0.0001	29.303	0.0001	19.268	0.0001
	Roots	266.737	0.0001	29.862	0.0001	21.827	0.0001
MG	Leaves	10.417	0.0047	13.386	0.0003	3.647	0.0468
	Roots	79.680	0.0001	10.300	0.0010	9.903	0.0013
CAT	Leaves	191.314	0.0001	9.637	0.0014	15.962	0.0001
	Roots	31.52	0.0001	3.777	0.0427	6.189	0.009
APX	Leaves	86.166	0.0001	12.93	0.0003	16.755	0.0001
	Roots	55.49	0.0001	4.058	0.0351	6.656	0.0069
DHAR	Leaves	25.309	0.0001	4.664	0.0233	5.473	0.0139
	Roots	37.051	0.0001	8.555	0.0024	3.833	0.0411
MDHAR	Leaves	26.007	0.0001	1.368	0.2800	15.693	0.0001
	Roots	66.874	0.0001	4.226	0.0313	7.493	0.0043
GR	Leaves	16.165	0.0008	2.043	0.1586	3.778	0.0426
	Roots	110.825	0.0001	1.723	0.2067	9.117	0.0018
GuPX	Leaves	217.195	0.0001	36.986	0.0001	51.185	0.0001
	Roots	54.656	0.0001	11.078	0.0007	4.057	0.0351
SOD	Leaves	12.32	0.0025	3.239	0.0629	4.848	0.0207
	Roots	41.531	0.0001	27.907	0.0001	31.707	0.0001
ATPS	Leaves	9.232	0.0071	5.500	0.0137	0.755	0.4843
	Roots	12.320	0.0025	3.239	0.0629	4.848	0.0207
APR	Leaves	113.997	0.0001	0.727	0.4968	5.031	0.0184
	Roots	23.398	0.0001	5.134	0.0172	0.800	0.4648
SiR	Leaves	4.874	0.0405	3.977	0.0371	3.959	0.0376
	Roots	192.939	0.0001	3.656	0.0465	2.181	0.1419
CS	Leaves	26.086	0.0001	1.280	0.3021	2.982	0.0761
	Roots	21.243	0.0002	3.901	0.0391	1.237	0.3138
γGCS	Leaves	94.341	0.0001	26.953	0.0001	14.875	0.0002
	Roots	51.319	0.0001	8.058	0.0032	4.799	0.0214
γGT	Leaves	28.689	0.0001	17.931	0.0001	0.015	0.9847
	Roots	0.227	0.6395	9.741	0.0014	7.478	0.0043
GS	Leaves	72.779	0.0001	5.858	0.011	9.099	0.0019
	Roots	61.490	0.0001	44.507	0.0001	10.545	0.0009
GST	Leaves	60.967	0.0001	6.964	0.0058	16.534	0.0001
	Roots	17.142	0.0006	0.827	0.4534	2.430	0.1164
Gly I	Leaves	6.486	0.0202	3.442	0.0542	11.448	0.0006
	Roots	15.094	0.0011	0.310	0.7371	4.427	0.0273
Gly II	Leaves	35.938	0.0001	5.164	0.0169	1.36	0.2817
	Roots	63.194	0.0001	4.415	0.0275	18.373	0.0001
TA	Leaves	52.03	0.0001	4.254	0.0307	3.536	0.0507
	Roots	7.208	0.0151	19.349	0.0001	21.677	0.0001
ASC	Leaves	51.188	0.0001	3.303	0.0600	2.444	0.1151
	Roots	4.307	0.0526	18.107	0.0001	19.886	0.0001
DHA	Leaves	46.189	0.0001	7.970	0.0033	8.222	0.0029
	Roots	16.138	0.0008	7.667	0.0039	10.461	0.0010
ASC/TA	Leaves	1.885	0.1866	3.859	0.0403	3.392	0.0562
	Roots	15.148	0.0011	7.229	0.005	6.852	0.0061
TG	Leaves	17.620	0.0005	17.317	0.0001	11.582	0.0006
	Roots	131.035	0.0001	5.465	0.0140	13.159	0.0003
GSH	Leaves	21.491	0.0002	17.710	0.0001	11.834	0.0005
	Roots	119.382	0.0001	5.077	0.0179	10.920	0.0008
GSSG	Leaves	0.829	0.3746	7.398	0.0045	4.942	0.0195
	Roots	4.528	0.0474	0.131	0.8779	1.935	0.1733

GSH/TG	Leaves	12.745	0.0022	7.042	0.0055	6.615	0.0070
	Roots	6.602	0.0193	3.248	0.0625	2.805	0.0870
MTs	Leaves	101.99	0.0001	6.290	0.0085	3.979	0.0371
	Roots	1.035	0.3224	28.990	0.0001	14.955	0.0001
PCs	Leaves	39.947	0.0001	1.498	0.2501	1.820	0.1906
	Roots	34.18	0.0001	10.549	0.0009	9.572	0.0015
TNP-SH	Leaves	41.041	0.0001	1.657	0.2184	1.891	0.1798
	Roots	34.879	0.0001	10.767	0.0008	9.804	0.0013
